# The dipteran family Celyphidae in the New World, with discussion of and key to world genera (Insecta, Diptera)

**DOI:** 10.3897/zookeys.711.20840

**Published:** 2017-10-23

**Authors:** Stephen D. Gaimari

**Affiliations:** 1 California State Collection of Arthropods, Plant Pest Diagnostics Center, California Department of Food & Agriculture, 3294 Meadowview Road, Sacramento, California 95832, USA

**Keywords:** Diptera, Lauxanioidea, new genus, new combination, new synonym, revised status, distribution

## Abstract

The family Celyphidae (Diptera, Lauxanioidea) is verified as part of the New World fauna, with a second specimen discovered of a species described from French Guiana in 1844 by P.J.M. Macquart. As this species possesses characteristics that clearly suggest a separate lineage from the Old World celyphids, a new genus is proposed, *Atopocelyphus* **gen. n.**, with the type species, *Celyphus ruficollis* Macquart, in the new combination *Atopocelyphus ruficollis* (Macquart), **comb. n.** A key to world genera of Celyphidae is presented, along with discussion of generic concepts. *Chamaecelyphus* Frey is synonymized under *Spaniocelyphus* Hendel, **syn. n.**, resulting in the following 10 new combinations: *Spaniocelyphus africanus* (Walker), **comb. n.**; *S. dichrous* (Bezzi), **comb. n.**; *S. gutta* (Speiser), **comb. n.**; *S. halticinus* (Frey), **comb. n.**; *S. kalongensis* (Vanschuytbroek), **comb. n.**; *S. ruwenzoriensis* (Vanschuytbroek), **comb. n.**; *S. straeleni* (Vanschuytbroek), **comb. n.**; *S. upembaensis* (Vanschuytbroek), **comb. n.**; *S. violaceus* (Vanschuytbroek), **comb. n.**; *S. vrydaghi* (Vanschuytbroek), **comb. n.** The subgenera of *Celyphus* Dalman are elevated to genus rank, as *Paracelyphus* Bigot, **stat. rev.**, and *Hemiglobus* Frey, **stat. rev.**, resulting in the following 17 new and revised combinations: *Hemiglobus cheni* (Shi), **comb. n.**; *H. eos* (Frey), **comb. n.**; *H. lacunosus* Frey, **comb. rev.**; *H. pellucidus* Frey, **comb. rev.**; *H. planitarsalis* (Shi), **comb. n.**; *H. porosus* (Tenorio), **comb. n.**; *H. pulchmaculatus* (Liu & Yang), **comb. n.**; *H. quadrimaculatus* (Tenorio), **comb. n.**; *H. resplendens* Frey, **comb. rev.**; *H. rugosus* (Tenorio), **comb. n.**; *H. testaceus* (Malloch), **comb. n.**; *H. trichoporis* (Shi), **comb. n.**; *H. unicolor* Frey, **comb. rev.**; *H. violaceus* Chen, **comb. rev.**; *Paracelyphus hyacinthus* Bigot, **comb. rev.**; *P. medogis* (Shi), **comb. n.**; *P. vittalis* (Shi), **comb. n.**

## Introduction

The Celyphidae is a small family in the Lauxanioidea (Diptera, Acalyptratae) characterized by their greatly enlarged scutellum and sharp reductions in chaetotaxy. Their gestalt is suggestive of certain metallic chrysomelid beetles. They are known to have their greatest diversity in tropical Asia and Southeast Asia, with a smaller number of species in the Afrotropical Region. The topic of this paper is one of the earliest described species in the group. The species *Celyphus ruficollis* Macquart, 1844 was the third species described in what is now the family Celyphidae, preceded only by *Celyphus obtectus* Dalman, 1818 and *Celyphus scutatus* Wiedemann, 1830. By the end of that century, an additional 14 species had been described (2 of them in an additional genus, *Paracelyphus* Bigot). Since that time, the family Celyphidae has grown to 115 valid species (of nearly 130 described) within 8 valid genera (of 9 described). Tenorio (1972) is the most comprehensive work on the family, although only dealing with the fauna of the Oriental Region, describing 21 new species-group taxa in addition to redescribing the then-known species in that region. Only 30 additional species have been described in the 45 years since that work.

**Figures 1–2.**
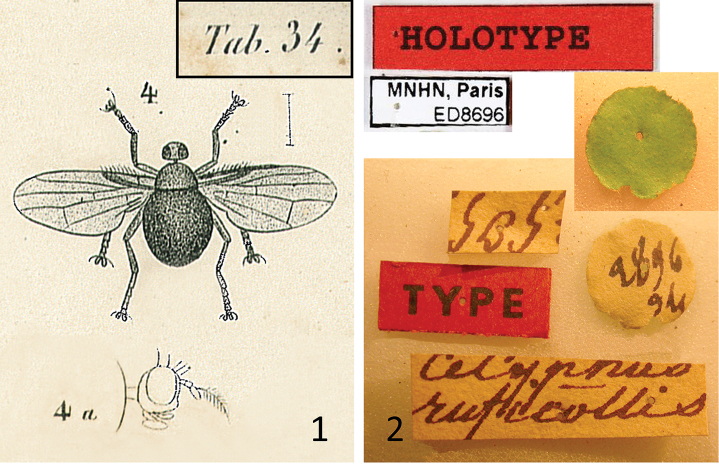
*Celyphus ruficollis* Macquart (=*Atopocelyphus ruficollis*), original materials. **1** Planche 34, figures 4 and 4a from Macquart (1844) **2** Labels on male syntype from MNHN (inset green circle is bottom of circular label).

After the original description by Macquart (1844), *Celyphus ruficollis* has been rarely mentioned in the literature, and only ever by repeating information from the original description. For example, Bigot (1878) included the species, along with its type locality, in a list of species included in the “*Celyphes*”. Later, in the catalog of celyphids authored by Jacobson (1896), this species is listed as “? *C. ruficollis*”, properly recording it from Guyana gallica (=French Guiana). Given the footnote for this entry (“*Secundum figuram cl. Macquarti haec species ob oculos haud prominentes aristamque aliter constructam genus peculiare, Paracelypho affine, constituere videtur*.” = According to the figure of Macquart this species has eyes that do not overhang the arista so is a different genus built more specifically akin to *Paracelyphus*), it seems his questioning its inclusion within *Celyphus* Dalman was only meant to suggest it may represent a different genus more similar to *Paracelyphus*. The following year, Wandolleck (1897) repeated the list of celyphid species as reported by Jacobson (1896). Later, Frey (1941) suggested that *Celyphus ruficollis* is likely not a celyphid due to the presence of fronto-orbital setae evident on plate 34, figure 4a of Macquart (1844) (Fig. 1), and afterwards, Vanschuytbroek (1952) did not mention the species when listing the species known at that time. Tenorio (1972), in her revision of Celyphidae of the Oriental Region, mistakenly referred to the species as having been described from Australia, and offered no further information. However, this was likely a mix-up with a different species, *Celyphus inaequalis* Costa, 1864, which was described from “Australia ?”, and is, like the current species under study, unknown after its initial description, with the family otherwise not known from the continent of its type locality.

The single syntype of *Celyphus ruficollis* was collected by François René Mathias Leprieur, during his time collecting in French Guiana (recorded by Macquart as “de la Guyane”). According to Papavero (1971), Leprieur spent much of his life in French Guiana, where he explored as an entomologist, including collections made in Cayenne, and a trip into the interior up the Oyapock River (which forms much of the border between French Guiana and Brazil) in 1832. Although some of his collections survived, much was lost in a shipwreck in 1833. In 1834, Leprieur donated at least 550 insect specimens, including this one, to the MNHN (see remarks below).

## Methods

The specimens examined of this New World celyphid were from two collections, as follows:

**BMNH** The Natural History Museum, London, England, United Kingdom.

**MNHN** Muséum national d’Histoire naturelle, Paris, France.

The specimens photographed of other genera of celyphids were from the following collections:

**CSCA** California State Collection of Arthropods, California Department of Food & Agriculture, Sacramento, California, USA.

**IZAS** Institute of Zoology, Academia Sinica, Beijing, China.

**USNM** National Museum of Natural History, Washington, DC, USA.

Morphological terminology follows Cumming and Wood (2009). In the description below, the state in the female is given in square brackets [ ] if different from the male, noting that having a single male and a single female for study, differences may be due to simple variation in the species or minor sexual dimorphism, or the remote possibility of being a different species.

## Taxonomy

### Atopocelyphus

Taxon classificationAnimaliaDipteraCelyphidae

Gaimari gen. n.

http://zoobank.org/74AF83BD-16AF-4489-95EE-66C67E0C37AD

#### Type species.

*Celyphus ruficollis* Macquart, 1844, by present designation.

#### Etymology.

From Greek, *Atopos*, meaning out of place, combined with the genus name *Celyphus*, referring to the unexpected occurrence of this taxon in the New World; masculine.

#### Diagnosis.

This genus differs from all other Celyphidae in having an elongate first flagellomere with a subbasal, plumose arista (Fig. 13), and in having abdominal tergites 5 and 6 each subdivided or creased medially with a strong triangular notch along each posterior edge in both sexes (Figs 18, 20).

#### Remarks.

The other celyphid genera have a much shorter first flagellomere with a subapical arista that is pubescent and often expanded and leaf-shaped in the basal 1/3 (see Fig. 29). The abdominal tergites are sometimes subdivided (i.e., in *Spaniocelyphus*), but this is always tripartite, with a central section and two lateral sections (Fig. 31); otherwise, the tergites are undivided (Fig. 28). With regards to other dipteran families in the Neotropics with superficially similar genera, *Celypholauxania* Hendel (Lauxaniidae) and *Peltopsilopa* Hendel (Ephydridae) share a characteristically enlarged scutellum, although none to the extent of the Celyphidae. One of the species currently in *Peltopsilopa* had been originally described as a species of *Celyphus* (Savaris et al. 2016), and other genera (outside the New World) had also been originally described as celyphids, such as *Afrocelyphus* Vanschuytbroek, now considered a junior synonym of *Nomba* Walker (Chloropidae).

### Atopocelyphus ruficollis

Taxon classificationAnimaliaDipteraCelyphidae

(Macquart)

Celyphus ruficollis Macquart, 1844: 253; Planche 34, figs 4, 4a.

#### Specimens examined.

***Type.*** French Guiana. 1 syntype male (Figs 4–5, 9–12, 17–18); MNHN: Specimen MNHN-ED-ED8696 (permalink http://coldb.mnhn.fr/catalognumber/mnhn/ed/ed8696), in the Macquart collection. Labels (Fig. 2; explanation below, in Remarks) as follows: 2896 / 34 [handwritten, circular label, pale green on opposite side]; 535 [handwritten]; Celyphus / ruficollis [handwritten]; TYPE [red label]; HOLOTYPE [red label]; MNHN, Paris / ED8696. Pinned through mesonotum; good condition (antennal flagellomeres both broken off, right fore tarsus broken off, lower third of the right wing missing, lower edge of left wing damaged).

**Figure 3.**
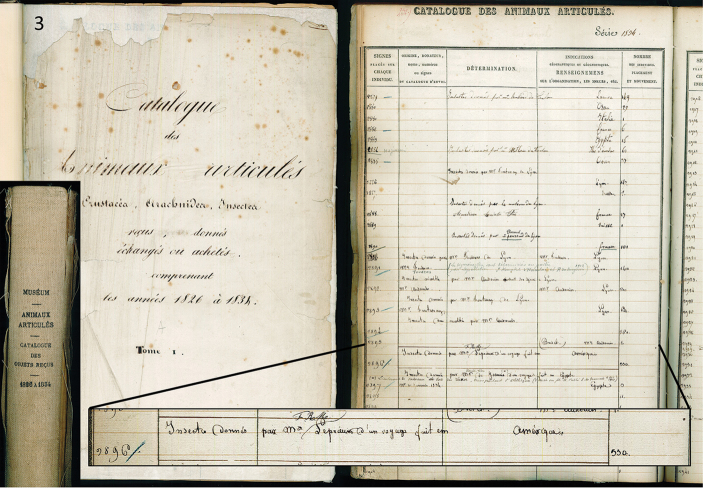
Catalogue des Animaux articulés Crustacéa, Arachnidea, Insectea, reçus, donnés échangés ou achetés comprenant les années 1826 à 1834. Tome I. **3** Spine (far left), front page (left side), page from the “Série 1834” (right side) containing the line for accession number 2896 (enlargement).

**Figures 4–5.**
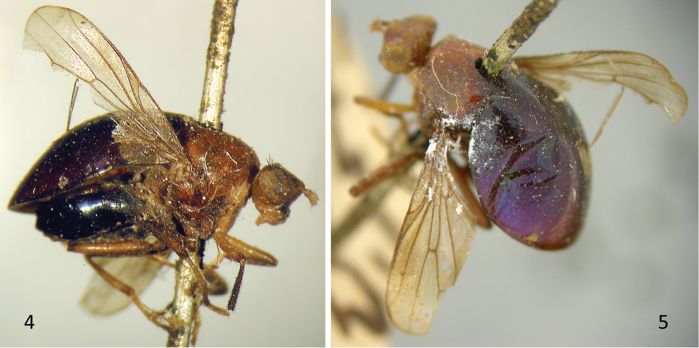
*Celyphus ruficollis* Macquart (=*Atopocelyphus ruficollis*), syntype male (MNHN). **4** Habitus, lateral **5** Habitus, dorsal oblique.

***Additional specimen.*** French Guiana: Réserve Trésor, xii.2009, Window trap, N 4°36'37.6" / W 52°16'44.5", altitude = ± 225 m. 1 female (Figs 6–8, 13–16, 19–20); BMNH, mounted on triangular point, scutellum removed and mounted on top of point; excellent condition.

**Figures 6–8.**
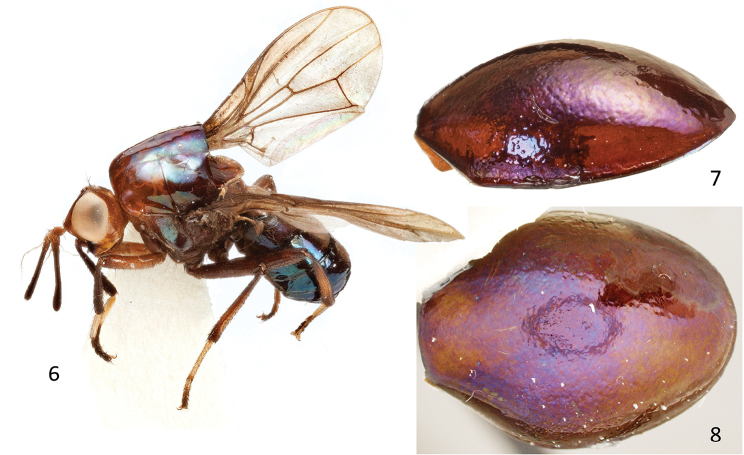
*Atopocelyphus ruficollis* (Macquart), female specimen (BMNH). **6** Habitus, lateral **7** Scutellum, lateral **8** Scutellum, dorsal.

#### Description.

Body (Figs 4–8) length (head through base of halter, plus base of halter through abdomen tip, to account for differential curling of the abdomen), [4.6–] 5.6 mm. Head through thorax length, including scutellum (head through posterior edge of scutum, plus scutellum), [6.2–] 6.4 mm. Head and thorax predominantly orange.

**Figures 9–12.**
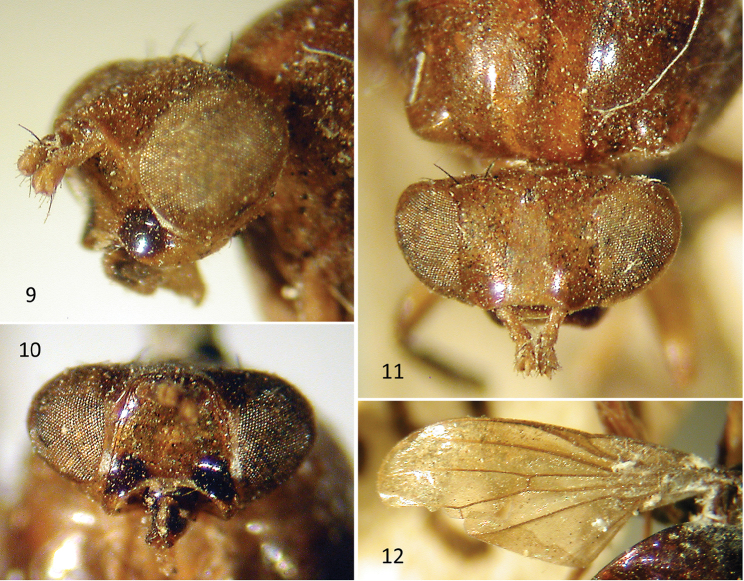
*Celyphus ruficollis* Macquart (=*Atopocelyphus ruficollis*), syntype male (MNHN). **9** Head, anterolateral. **10** Head, anteroventral **11** Head, dorsal. **12** Wing.

**Figures 13–16.**
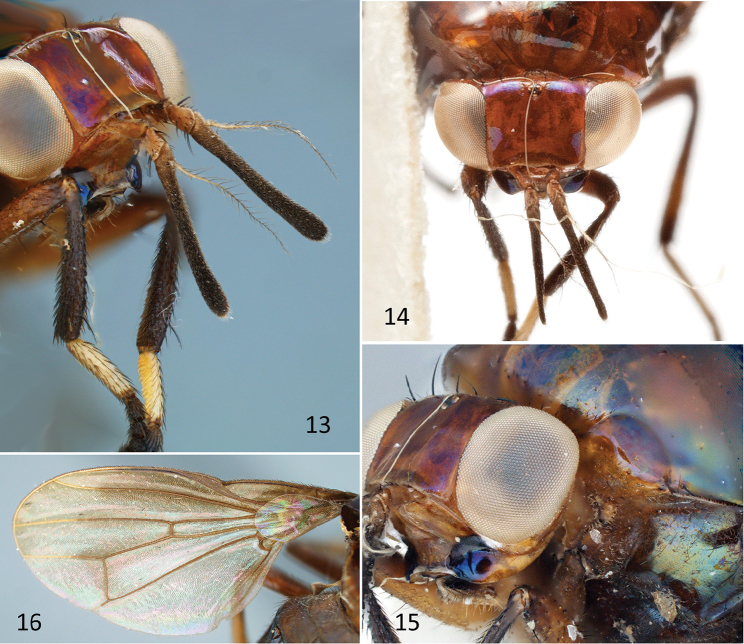
*Atopocelyphus ruficollis* (Macquart), female specimen (BMNH). **13** Head and forelegs, anterolateral **14** Head, dorsal **15** Head and anterior part of pleuron, anterolateral **16** Wing.

***Head*** (Figs 9–11, 13–15). Head length (excluding antennae) [0.9–] 1.1 mm, height [1.1–] 1.2 mm, width [1.6–] 1.8 mm; 1.6 [–1.8] × wider than long, 1.5 X wider than high. Vertex rounded; inner vertical seta incurved, 0.25 mm; outer vertical seta outcurved, 0.2 mm; postocellar setae cruciate, 0.2 mm, thinner than vertical setae; distance between inner and outer vertical setae subequal to distance between outer vertical seta and postocellar seta. Ocellar triangle equilateral, with distance from one ocellus to another 0.1 mm. Frons length (anterior ocellus to lunule) [0.55–] 0.65 mm, width [0.8–] 0.9 mm parallel sided, extending [0.16–] 0.18 mm anteriorly beyond edge of eye. Median vitta visible as roughened texture relative to fronto-orbital area being smooth and shiny (in holotype, only visible from dorsolateral aspect); width at ocellar triangle 0.2 mm, expanding anteriorly to 0.25 mm at lunule. Antennae (Figs 13–14) separated by [2.5–] 3 × width of an antennal base, rounded between antennae with no facial keel; scape and pedicel orange; scape length [0.15–] 0.25 mm, widening distally, fully exposed; pedicel 0.18 [–0.2] mm, with enlarged dorsal seta and 2 slightly enlarged ventral setae. Antennal first flagellomere black except orange basally to aristal base (broken off in holotype); length 1.05 mm; height 0.1 mm, slightly expanded distally to 0.15 mm at tip. Arista orange, becoming slightly darker distally; length 0.9 mm, not extended beyond tip of first flagellomere; plumose, with rays up to 0.15 mm. Face flat in upper 2/3, descending sharply from plane of frons; lower 1/3 of face + subgena recurved anteriorly. Gena narrow, with white pruinescence at interface with parafacial; 2 small, fine genal setulae (not evident in holotype). Subgena larger than gena, and bulging; with dark brown spot confluent with dark brown spot at lower corner of face. Clypeus narrow, dark brown ventrally. Palpus black, yellow basally, slightly flattened and spatulate, fuzzy and with several longer thin setulae.

***Thorax*** (Figs 4–6, 15). Scutum dorsal length 1.5 mm, width at suture 2.0 mm; lateral length (anterior edge to halter) [2.0–] 2.2 mm. Scutellum (Figs 5, 7–8) length [3.8–] 4.2 mm, width [2.8–] 3.4 mm, height [1.3–] 1.5 mm, extending beyond apex of abdomen (Fig. 4); concave ventrally, with sharpened ventral edge; dorsal surface smooth with irregular dimpling; ventrally hairy anterolaterally, with tiny hairs scattered throughout venter. Postpronotum with 1 small postpronotal seta, otherwise bare dorsally, setulose ventrolaterally; patch of small black setulae medial to postpronotum on anterior surface of mesonotum. Proepisternal seta present, short and fine. Prosternum orange, lightly fuzzy, but lacking setae or setulae. Mesonotum with dorsocentral setae 1 + 3, small and hair-like; with smaller and hair-like acrostichal setulae (1–2 presutural, 3 or 4 postsutural), with prescutellar pair slightly thickened (obscured by pin in holotype); 1 strong supra-alar seta above wing base; 2 fine postalar setae present; notopleuron with 2 black setae, posterior one stronger than anterior. Anepisternum and katepisternum orangish brown and with sparse whitish setulae; posterior margin of anepisternum with short black anepisternal seta (slightly longer than inner vertical seta); upper margin of katepisternum with 2 subequal, short, fine black katepisternal setae (broken off in holotype).

***Wing*** (Figs 12, 16). Wing length [4.2–] 4.5 mm, height [2.0–] 2.1 mm; sapromyziform, with spinules on costa ending at tip of R_2+3_; hyaline, but darkened brown basally and within costal cell, subcostal cell, and along costal vein to point between apices of R_1_ and R_2+3_, along vein R_2+3_ except apical 1/4, and with some slight darkening on R_4+5_ and crossvein r-m; veins brown except yellow on distal half of costal vein and distal parts of veins R_2+3_, R_4+5_ and M_1_; costal vein ends at apex of M_1_; crossvein r-m slightly beyond midpoint of discal cell; CuA_1_ short, 1/5 length of crossvein dm-cu, not reaching wing margin; A_1_+CuA_2_ short; A_2_ present only as darkened fold. Halter brown.

***Legs.*** Legs orangish brown, except as noted. Fore coxa orange; femur yellow orange, becoming dark brown distally, with 1 strong preapical posteroventral seta and row of [4–] 5 long thin posterodorsal setae; fore tibia dark brown, with 1 apicoventral spur and 1 strong preapical dorsal seta; fore tarsus with tarsomere 1 white, slightly longer than tarsomeres 2–5 combined, ventrally with dense pad of thickened orange yellow setulae; tarsomeres 2–5 dark brown. Mid coxa dark brown; mid femur setulose, but with no outstanding setae or setal rows; mid tibia with basal and apical parts dark brown, with 1 apicoventral spur and one strong preapical dorsal seta; mid tarsus with tarsomeres 1–2 yellow, tarsomeres 3–5 brown, ventrally with dark brown setulae. Hind coxa dark brown; hind femur setulose, but with no outstanding setae or setal rows; hind tibia with basal and apical parts dark brown, lacking apicoventral spur and preapical dorsal seta, but inner edge of apex with tight comb of yellowish brown setulae; hind tarsus with tarsomeres 1–2 pale yellow, tarsomeres 3–5 light brown, tarsomere 1 ventrally with dense pad of thickened orange yellow setulae, ventral setulae of remaining tarsomeres dark brown.

**Figures 17–18.**
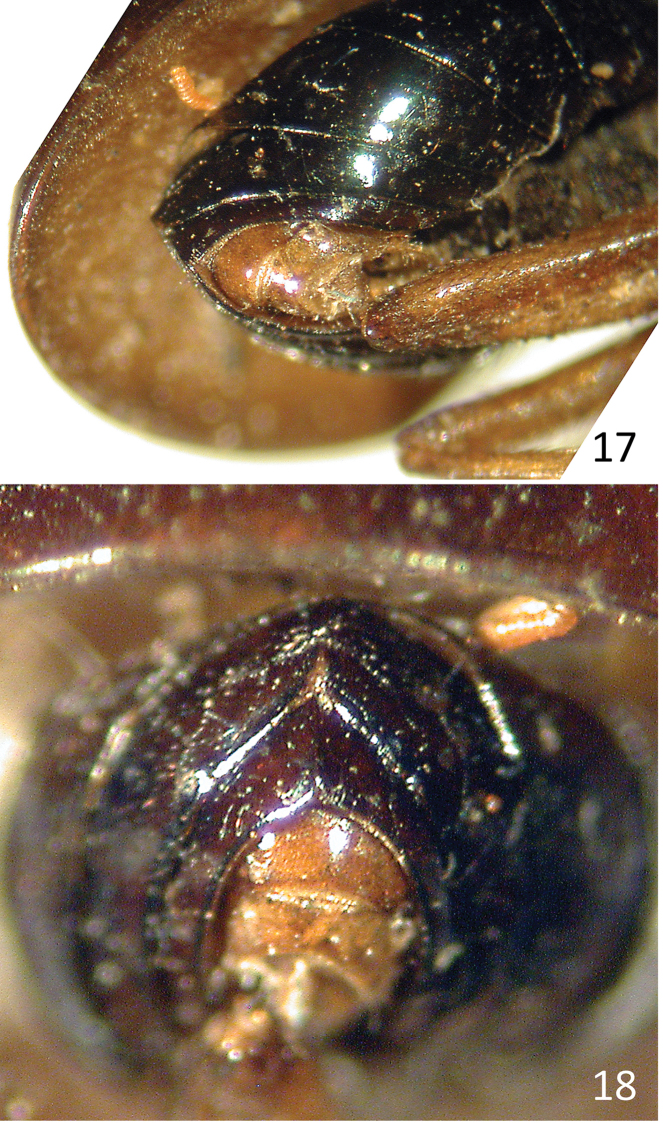
*Celyphus ruficollis* Macquart (=*Atopocelyphus ruficollis*), syntype male (MNHN). **17** Abdomen and genitalia, ventrolateral **18** Abdomen, posterior.

**Figures 19–20.**
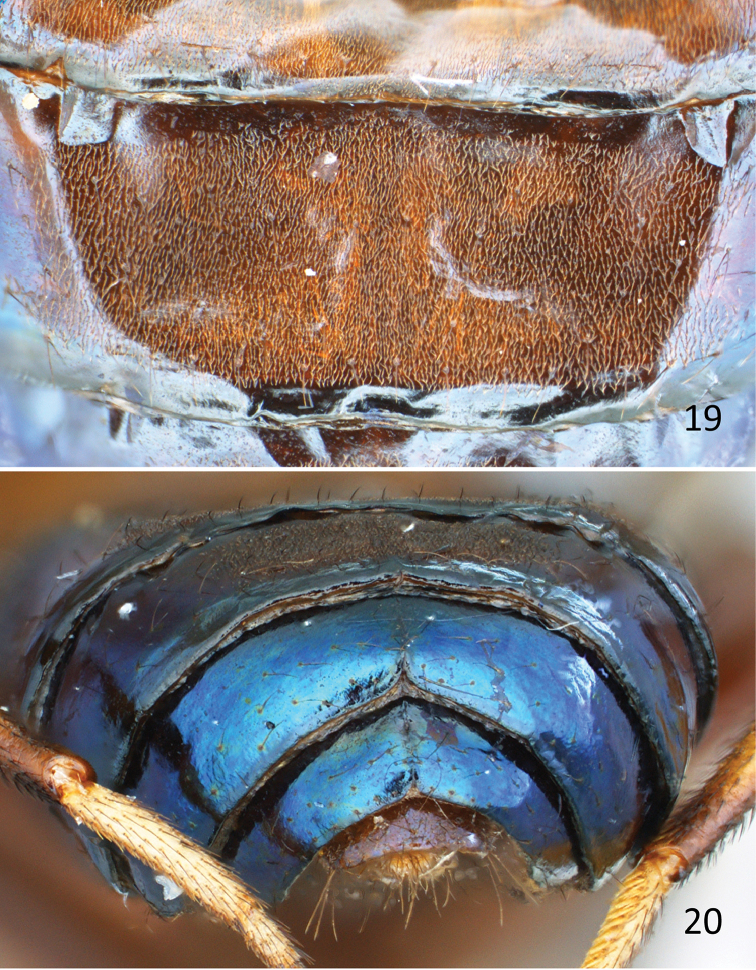
*Atopocelyphus ruficollis* (Macquart), female specimen (BMNH). **19** Abdomen, tergite 3, dorsal **20** Abdomen, posterodorsal.

***Abdomen*** (Figs 17–20). Abdomen length [2.2 –] 2.5 mm, width [1.8 –] 1.9 mm. Syntergite 1+2 and tergites 3 and 4 flattened (top part of tergites 3 and 4 of holotype missing due to dermestid damage), brown pruinose dorsally (Fig. 19) and shiny dark brown to blackish blue laterally; tergites 5 and 6 shiny dark brown to blackish blue (depending on angle of view); tergite 7 light brown. Tergites recurved laterally underneath abdomen, such that lateral parts visible from below; sparsely setulose, mostly smooth; tergite 4 with slight medial notch along posterior edge; tergites 5 and 6 each subdivided or creased medially, with strong triangular notch along posterior edge (Figs 18, 20); tergite 7 saddle-shaped. Sternites brown, irregularly hairy and with sparse setulae; each sternite wider than long.

***Male terminalia*** (Fig. 17). Epandrium saddle-shaped, about as long as wide, concolorous and fitting easily within saddle-shaped tergite 7; surstylus small, bilobed distally, hairy on lobes (no other characteristics visible without dissection).

***Female terminalia.*** Hypoproct orange, longer than wide, rounded distally, covered with pale brown setulae. Epiproct brownish, short, rounded distally, covered with dark setulae. Cerci orange, slightly longer than wide, with mixed pale and dark brown setulae, a few elongate.

#### Remarks.

Crosskey (1971) discusses in detail the labeling standards in the Macquart collection, which are directly applicable to this specimen (Fig. 2). The circular label is green on one side, meaning it is from the Americas, and the handwritten number on the white side is the accession number, which represents the MNHN serial number given to the collection to which the specimen formed a part, and the year of accession, in this case 2896 / 34 (serial number 2896, year of accession 1834). This accession number is found in the accessions book at the MNHN (Fig. 3), titled “Catalogue des Animaux articulés Crustacèa, Arachnidea, Insectea, reçus, donnés échangés ou achetés comprenant les années 1826 à 1834. Tome I.” Within this catalogue is the line for accession number 2896 in the “Série 1834”, as follows (columns separated by “/”): Insectes donnés / par M^r^ F.R.M. Leprieur d’un voyage fait en / Amérique / 550 [specimens]. As discussed by Crosskey (1971), Macquart’s type labels did not consistently indicate “n.sp.” (or similar) at the time of this publication, although he did start to consistently use this term on his labels after this time.

Besides these generalities of labeling of Macquart types, Pont (2012) also dealt with specimens of Leprieur from French Guiana in the MNHN, which, along with discussion with Adrian Pont, significantly aided my current interpretations of the labels on the type. The accession number 2896 refers to Leprieur’s collections in French Guiana. Noting that the label is slightly ripped at the bottom of the first number of the year, it is likely that this was accessioned in 1834, which is consistent with the types of the muscids *Limnophora elegans* Macquart and *Spilogaster maculipennis* Macquart, both collected by Leprieur “de la Guyane” from the same publication (Macquart, 1844). There is also reference in Pont (2012), but not Crosskey (1971), to an old handwritten 3 digit number label for the two preceding species, and the type of *Celyphus ruficollis* similarly has such a number label, 535. The handwriting on these labels is clearly that of Macquart, in comparison with labels presented by Crosskey (1971) and Pont (2012), and other Macquart specimens in the MNHN seen by the author. It is possible that this number represents a sort of “unique identifier” of the time, given that the Catalogue indicates 550 specimens were donated (i.e., that this was specimen 535 of 550). Another alternative is that the number was a reference to the species itself (i.e., that this number was a reference to Macquart’s notes on this species, although no such notes have been located). In any case, the meaning of this secondary number remains a mystery.

It is worth noting that Macquart’s description at least partly contradicts his figure 4a (plate 34) (Fig. 1), in that the description states that the arista is inserted near the tip (i.e., subapical, as is typical of all other celyphids), while the figure shows the arista as clearly subbasal. Unfortunately, the type specimen has lost the first flagellomere and arista, and given that the condition of these structures is important to the definition of this new genus, a further comment is warranted. In the newly collected specimen, the antennae are quite elongate, well beyond that of any other Celyphidae, and the arista is plumose and placed subbasally, all unique states in this species relative to other celyphids. Macquart (1844) does not mention an elongate antenna, and his figure 4a (plate 34) (Fig. 1) does not show an antenna of such length, but it does show the subbasal placement and the plumose condition of the arista, so it remains a possibility that the first flagellomere itself was broken (i.e., appearing short) when Macquart examined the specimen, and only later completely broke off and was lost. Also note, neither the type specimen nor the new specimen possess what appears to be the fronto-orbital setae pictured in figure 4a (plate 34) (Fig. 1). This is significant because Frey (1941) specifically refers to this figure to point out that this species is likely not a celyphid due to the presence of these setae.

### Key to world genera of Celyphidae

**Table:** 

1	Scutellum and mesonotum subequal in length (Fig. 21); mesonotum and scutellum with strong setae (e.g., dorsocentral, postpronotal, scutellar) (Fig. 21); hind tibia with a long, strong apical spur (Fig. 22)	***Idiocelyphus* Malloch**
–	Scutellum longer than mesonotum (Figs 23–25); setae of mesonotum tiny or absent and scutellum lacking strong setae (Figs 23–25); hind tibia lacking an apical spur	**2**
2	Body stout (Figs 24–25); vertex rounded (Figs 33–34)	**4**
–	Body elongate (Fig. 23); vertex carinate (Figs 26, 29)	**3**
3	Postocellar setae strong, convergent (Fig. 26); palpus broadly spatulate at apex (Fig. 27); abdominal tergites lacking longitudinal sutures (Fig. 28)	***Acelyphus* Malloch**
–	Postocellar setae tiny, hair-like (Fig. 29); palpus cylindrical (Fig. 30); abdominal tergites with dorsolateral longitudinal sutures dividing each into three sections (Fig. 31)	***Spaniocelyphus* Hendel**
4	First flagellomere elongate (Fig. 13); arista plumose placed subbasally on first flagellomere (Fig. 13); abdominal tergites 5 and 6 each subdivided or creased medially with a strong triangular notch along each posterior edge (Figs 18, 20)	***Atopocelyphus* gen. n.**
–	First flagellomere not elongate, at most subequal to pedicel plus scape length; arista pubescent (Fig. 34), or broadly flattened in basal part (Fig. 33), placed subapically on first flagellomere; abdominal tergites undivided	**5**
5	Basal tarsomere on fore and hind tarsus (and sometimes mid tarsus) angularly produced at the outer side near the base (Fig. 32)	***Oocelyphus* Chen**
–	Basal tarsomeres with no angularly produced areas	**6**
6	First flagellomere tapering distally, 2 times longer than high (Fig. 33); arista broadly flattened and leaf-like in at least basal 1/3 (Fig. 33)	***Celyphus* Dalman**
–	First flagellomere rounded distally, at most 1.5 times longer than high (Fig. 34); arista setaceous and pubescent (Fig. 34)	**7**
7	Scutellum with distinct lateral furrow (Fig. 36); ovoid and slightly tapering posteriorly (Fig. 25)	***Paracelyphus* Bigot, stat. rev.**
–	Scutellum lacking lateral furrow (Fig. 35), broadly rounded posteriorly (as in Fig. 24)	***Hemiglobus* Frey, stat. rev.**

**Figures 21–22.**
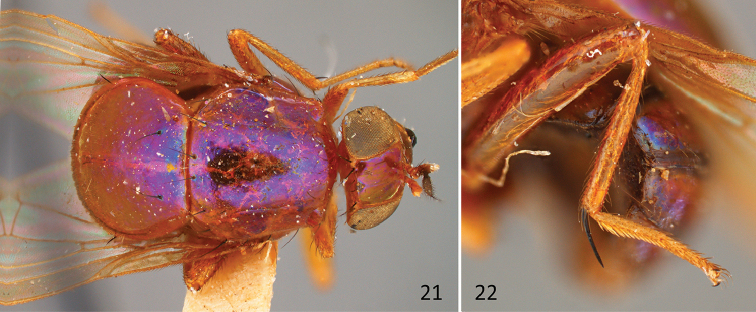
*Idiocelyphus bakeri* Malloch, PT female (USNM; Philippines). **21** Habitus, dorsal **22** Hind leg.

**Figures 23–25.**
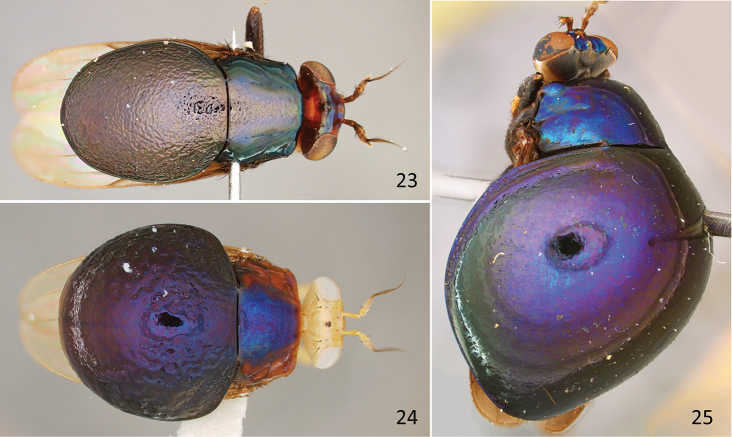
Habitus, dorsal. **23** *Spaniocelyphus cognatus* Karsch, female specimen (USNM; India) **24** *Celyphus aurora* Karsch, female specimen (USNM; Thailand) **25** *Paracelyphus hyacinthus* Bigot, male specimen (USNM; Malaysia), slightly dorsolateral.

**Figures 26–31.**
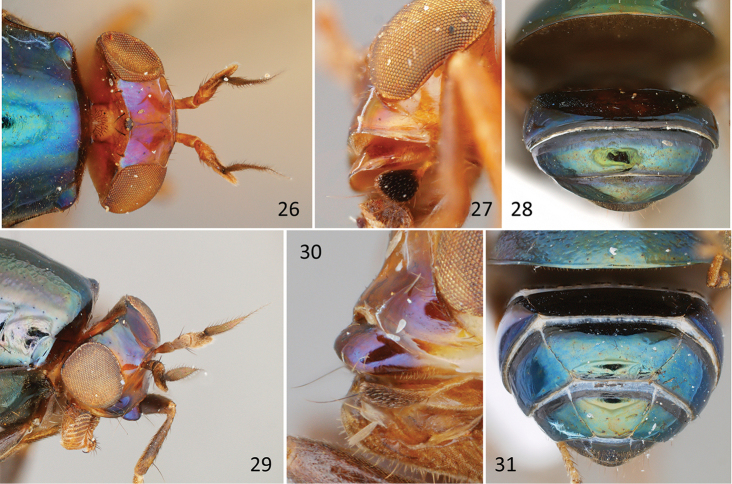
*Acelyphus* and *Spaniocelyphus*. **26** *Acelyphus repletus* Malloch, PT male (USNM; Singapore), head and anterior thorax, dorsal **27** *Acelyphus politus* Malloch, PT female (USNM; Philippines), lower part of head and palpus, lateral **28** *Acelyphus repletus* Malloch, female specimen (CSCA; Malaysia), abdomen, dorsal **29** *Spaniocelyphus cognatus* Karsch, female specimen (USNM; India), head and anterior thorax, dorsolateral **30** *Spaniocelyphus cognatus* Karsch, male specimen (USNM; India), lower part of head and palpus, lateral **31** *Spaniocelyphus palmi palmi* Frey, male specimen (CSCA; Malaysia).

**Figures 32–36.**
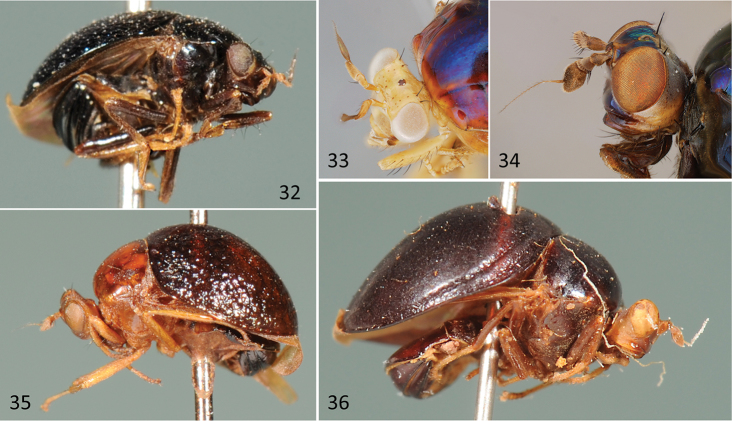
*Oocelyphus*, *Celyphus*, *Paracelyphus*, *Hemiglobus*. **32** *Oocelyphus nigritus* Shi, HT male (IZAS; China), habitus, ventrolateral **33** *Celyphus aurora* Karsch, female specimen (USNM; Thailand), head and anterior thorax, dorsolateral **34** *Paracelyphus hyacinthus* Bigot, male specimen (USNM; Malaysia), head, lateral **35** *Hemiglobus violaceus* Chen, HT male (IZAS; Vietnam), habitus, lateral **36** *Paracelyphus vittalis* (Shi), HT female (IZAS; China), habitus, lateral.

## Remarks

In their treatments of Celyphidae, Frey (1941), who described *Chamaecelyphus*, and Vanschuytbroeck (1952, 1953, 1959, 1963), differentiated *Chamaecelyphus* from *Spaniocelyphus* based on the absence or presence of the bm-cu crossvein on the wing. At that time, geography also separated these genera, with *Chamaecelyphus* being restricted to the Afrotropics and *Spaniocelyphus* being from the Oriental Region. Stuckenberg (1960) described two species that had a faint bm-cu crossvein, recording *Spaniocelyphus* for the first time in the Afrotropics, and pointing out that the grounds for separating these two genera are very slight. With examination of numerous specimens from the Afrotropics, by Ray Miller and me, we have seen many series where the bm-cu crossvein is absent or present (even faintly) within the same species, concluding that this is not a consistent character, and is certainly not a good basis for separating genera. As such, *Chamaecelyphus* is herein synonymized under *Spaniocelyphus*, syn. n., resulting in the following 10 new combinations (original genus *Chamaecelyphus* unless otherwise indicated): *Spaniocelyphus africanus* (Walker, 1849; *Celyphus*), comb. n.; *S. dichrous* (Bezzi, 1908; *Celyphus*), comb. n.; *S. gutta* (Speiser, 1910; *Celyphus*), comb. n.; *S. halticinus* (Frey, 1941), comb. n.; *S. kalongensis* (Vanschuytbroek, 1963), comb. n.; *S. ruwenzoriensis* (Vanschuytbroek, 1963), comb. n.; *S. straeleni* (Vanschuytbroek, 1959), comb. n.; *S. upembaensis* (Vanschuytbroek, 1952), comb. n.; *S. violaceus* (Vanschuytbroek, 1959), comb. n.; *S. vrydaghi* (Vanschuytbroek, 1952), comb. n.

The genus-group taxa *Paracelyphus* and *Hemiglobus* have been considered as separate full genera or as subgenera of *Celyphus*. Authors since Tenorio (1972) have followed the latter scheme, although her justification did not take into account the genus *Oocelyphus* since she had never studied species in this genus. When considering the four “*Celyphus*-like” genus-group taxa (i.e., the stout-bodied genera, as in Fig. 24), there is no reason to infer that *Celyphus* forms a natural group with *Hemiglobus* and *Paracelyphus* to the exclusion of *Oocelyphus*. In fact, *Oocelyphus* shares the expanded and leaf-like arista, and the more elongate and tapering first flagellomere, found in *Celyphus*, and appears more generally similar to *Celyphus* than either of the other two genera. *Celyphus* and *Oocelyphus* are easily separated by the expanded and angularly produced basal tarsomeres in the latter genus. Both of the other genera are larger-bodied (especially *Paracelyphus*, but also some *Hemiglobus*), and share a setaceous arista. They are easily separated from each other by the presence of a lateral scutellar furrow and a posteriorly tapering scutellum in *Paracelyphus*. As such, these two genus-group taxa are removed from synonymy under *Celyphus* (as subgenera), and instead recognized at full genus rank, resulting in the following 17 new and revised combinations: *Hemiglobus cheni* (Shi, in Liu et al. 1998; *Celyphus*), comb. n.; *H. eos* (Frey, 1941; *Celyphus*), comb. n.; *H. lacunosus* Frey, 1941, comb. rev.; *H. pellucidus* Frey, 1941, comb. rev.; *H. planitarsalis* (Shi, in Liu et al. 1998; *Celyphus*), comb. n.; *H. porosus* (Tenorio, 1972; *Celyphus*), comb. n.; *H. pulchmaculatus* (Liu & Yang, in Yang and Liu 2002; *Celyphus*), comb. n.; *H. quadrimaculatus* (Tenorio, 1972; *Celyphus*), comb. n.; *H. resplendens* Frey, 1941, comb. rev.; *H. rugosus* (Tenorio, 1972; *Celyphus*), comb. n.; *H. testaceus* (Malloch, 1929; *Paracelyphus*), comb. n.; *H. trichoporis* (Shi, in Liu et al. 1998; *Celyphus*), comb. n.; *H. unicolor* Frey, 1941, comb. rev.; *H. violaceus* Chen, 1949, comb. rev.; *Paracelyphus hyacinthus* Bigot, 1859, comb. rev.; *P. medogis* (Shi, in Liu et al. 1998; *Celyphus*), comb. n.; *P. vittalis* (Shi, in Liu et al. 1998; *Celyphus*), comb. n.

## Supplementary Material

XML Treatment for Atopocelyphus

XML Treatment for Atopocelyphus ruficollis
